# Suppressive Effects of Hainosan (Painongsan) against Biofilm Production by *Streptococcus mutans*

**DOI:** 10.3390/dj8030071

**Published:** 2020-07-06

**Authors:** Masaaki Minami, Hiroshi Takase, Masayo Taira, Toshiaki Makino

**Affiliations:** 1 Department of Bacteriology, Graduate School of Medical Sciences, Nagoya City University, 1 Kawasumi, Mizuho-ku, Nagoya 467-8601, Japan; 2 Core Laboratory, Graduate School of Medical Sciences, Nagoya City University, Nagoya 467-8601, Japan; takase@med.nagoya-cu.ac.jp; 3 JPS Pharmaceutical Co. Ltd., 4-42-22 Higashiyamata, Tsuzuki-ku, Yokohama 224-0023, Japan; m-taira@jps-pharm.com; 4 Department of Pharmacognosy, Graduate School of Pharmaceutical Sciences, Nagoya City University, 3-1 Tanabe-Dori, Mizuho-ku, Nagoya 467-8601, Japan; makino@phar.nagoya-cu.ac.jp

**Keywords:** biofilm, Hainosan, Japanese traditional Kampo medicine, *Platycodon grandiflorum*, *Streptococcus mutans*

## Abstract

*Streptococcus mutans*, a bacterium that causes dental plaques, forms a biofilm on tooth surfaces. This biofilm can cause gingivitis by stimulating the gingival margin. However, there is no established treatment for biofilm removal. Hainosan (Painongsan), a traditional Japanese Kampo formula, has been used to treat gingivitis. Therefore, we investigated the biofilm suppressive effects of the hainosan extract (HNS) and its components on *S. mutans*. We conducted scanning electron microscopy and confocal laser microscopy analyses to clarify the anti-biofilm activities of HNS and its crude drugs. We also performed a quantitative RT-PCR assay to assess the biofilm-related gene expression. HNS showed a significant dose-dependent suppressive effect on biofilm formation. Both the scanning electron microscopy and confocal laser microscopy analyses also revealed the significant inhibitory effects of the extract on biofilm formation. Transmission electron microscopy analysis showed that HNS disrupted the surface of the bacterial wall. Furthermore, HNS reduced the hydrophobicity of the bacteria, and suppressed the mRNA expression of β-glucosyltransferase (*gtfB*), glucosyltransferase-SI (*gtfC*), and fructosyltransferase (*ftf*). Among the constituents of hainosan, the extract of the root of *Platycodon grandiflorum* (PG) showed the strongest biofilm suppression effect. Platycodin D, one of the constituent natural compounds of PG, inhibited *S. mutans*-associated biofilm. These findings indicate that hainosan eliminates dental plaques by suppressing biofilm formation by *S. mutans*.

## 1. Introduction

Dental plaques induce dental caries and periodontal disorders [1]. The proliferation of dental plaques will lead to dental decay [2]. As the bacterial degradation of fermentable sugar produced acid, plaques cause the localized ablation of the tooth tissues. This also leads to periodontal troubles like periodontitis and gingivitis [3]. Dental plaque is an oral bacterial biofilm [4]. This plaque is commonly confirmed around the teeth and the gum line, constituting a microbial community of a particular structure with a specific function [5]. Salivary pellicles which act as an adhesive may immediately colonize a clean tooth surface. This permits the early colonizers to adhere to the tooth. These early colonizers are mainly Streptococcus species such as *Streptococcus mutans* [6].

*S. mutans* is regarded as a primal dental cariogenic bacterium [7]. It has some virulence factors that are linked to its cariogenicity [6]. The central virulence characteristic of *S. mutans* is its capacity to metabolize various sugar carbohydrates [5]. *S. mutans* uses this ability to form biofilms on the enamel surface of the tooth [5]. Bacterial biofilm is mostly composed of bacterial micro-colonies in a polymeric extracellular matrix [7]. This matrix blocks mechanical removal and host protein interactions. Furthermore, it acts as a diffusion barrier against several antimicrobial agents [5,6,7].

Among the most successful strategies for the discovery of novel agents is the usage of natural products derived from medicinal plants [8]. Because *S. mutans* plays a major role in dental decay, it should be removed as much as possible [5]. Thus, some strategy about the reduction of count and activity of *S. mutans* is valuable for maintaining a healthy oral cavity [3]. It has already been mentioned that plant extracts and their components possess drastic antimicrobial actions on oral bacteria, particularly *S. mutans* [9]. Several phytochemically rich extracts and their compounds have shown the suppressive effects of biofilm formation with the attachment of *S. mutans* [9].

Hainosan (Painongsan) is a formula used in traditional Chinese medicine and traditional Japanese Kampo medicine. The prescription of hainosan was described in Jingui Yaolüe by Zhang Zhongjing in the Later Han Dynasty. This formula has been explained for the treatment of acute or chronic purulent diseases, including periodontal disease. Hainosan contains three kinds of herbs: the dried immature fruit of *Citrus aurantium* (Aurantii Fructus Immaturus, CA), the dried root of *Paeonialactiflora* (Paeoniae Radix, PL), and the dried root of *Platycodon grandiflorum* (Platycodon Radix, PG) [10].

While we found that the hainosan extract (HNS) may suppress the bacterial growth and biofilm formation of *Porphyromonas gingivalis* [11,12], it remains unclear whether HNS is effective against another oral pathogenic bacterium, *S. mutans*. Thus, our objective was to investigate the antibacterial effects of HNS against *S. mutans* in this study.

## 2. Materials and Methods

### 2.1. Crude Drugs and Extract Preparation

Hainosan consists of 2.25 g of CA, 2.25 g of PL, 1.125 g of PG in a daily dose for humans. Each crude drug was supplied from JPS Pharmaceutical. Co. Ltd. (Yokohama, Japan). These crude drugs were under the grade of Japanese Pharmacopoeia 17th Edition [13]. A four-day dose of hainosan (total 22.5 g) or each crude drug (10 g) was boiled in water (20 times the weight) for 30 min. After filtering, the decoctions were lyophilized. Finally, the dried powdered extracts were yielded (ratio of the yielded extract to the original weights of crude drug(s): 34.6% of HNS for hainosan, 40.3% for CA, 43.7% for PL, and 57.3% for PG). Each extract was suspended in H_2_O and stored at −20 °C until use. The fingerprint image of HNS was presented elsewhere [11].

### 2.2. Bacterial Strains

*S. mutans* UA159 (ATCC700610, Manassas, VA, USA) was used in this study [7]. A bacterial colony was inoculated on trypticase soy agar with 5% sheep blood (Becton Dickinson, Franklin Lakes, NJ, USA) and cultured for 24 h at 37 °C under 5% CO_2_. The bacteria were suspended in sterile PBS again. The bacterial density was defined by quantifying the absorbance at 600 nm (A600). The bacterial suspension was diluted with PBS to 10^8^ CFU (colony forming unit)/mL.

### 2.3. In Vitro Growth Study

*S. mutans* (1 × 10^6^ CFU) were cultured in trypticase soy broth (Becton Dickinson) with 0.25% sucrose (Fujifilm Wako Pure Chemical, Osaka, Japan) (TSB) with the HNS extract at 37 °C under 5% CO_2_ for 24 h. After that, the aliquot was cultured on trypticase soy agar with 5% sheep blood agar at 37 °C for 24 h under 5% CO_2_.

### 2.4. Safranin Red Staining

We performed the biofilm assay with some modification [12]. *S. mutans* (1 × 10^6^ CFU) were cultured TSB in 96-well polystyrene plates (Thermo Fisher Scientific, Waltham, MA, USA) with or without HNS, the extract, or platycodin D (Fujifilm Wako) for 48 h at 37 °C. After the removal of the medium, we washed the plates three times with PBS. After that, the attached bacteria were stained with 0.2% safranin red (Fujifilm Wako) for 10 min at room temperature and gently washed three times with PBS. Each biofilm was evaluated by quantifying the absorbance at 490 nm. Wells incubated without bacteria were used as blanks. Absorbance of the blank wells was subtracted from the test values.

### 2.5. Confocal Laser Microscopy

We performed a confocal microscopic analysis with minor modifications [14]. *S. mutans* was cultured in TSB on glass coverslips placed in 24-well polystyrene plates (Thermo Fisher) for 48 h at 37 °C under 5% CO_2_. After the removal of the medium, we washed the wells three times with PBS. We stained the glass coverslips with fluorescein isothiocyanate isomer (FITC) (Fujifilm Wako). The biofilm images were evaluated utilizing an LSM 510 confocal laser microscope (Carl Zeiss, Oberkochen, Germany). We also created three-dimensional images from Z-stack images by utilizing Imaris software (Carl Zeiss).

### 2.6. Scanning Electron Microscopic (SEM)

We carried out the preparation for the scanning electron microscopic analysis as described earlier [14]. *S. mutans* treated with HNS and those not treated with HNS were incubated in TSB on glass coverslips in 24-well polystyrene plates (Thermo Fisher) for 48 h at 37 °C under 5% CO_2_. Subsequently, we placed the coverslips in 2.5% glutaraldehyde (Nisshin EM, Tokyo, Japan) prepared in 0.1 M phosphate buffer (pH 7.4) at 4 °C for 24 h. After the coverslips were rinsed 2 times with 0.1 M phosphate buffer (pH 7.4), we post-fixed the coverslips utilizing 2% osmium tetroxide (Nisshin EM) at 25 °C for 2 h. We rinsed with distilled water finally. Then, we dehydrated the samples utilizing graduated concentrations of ethyl alcohol for 30 min each. The samples were dried using a critical point dryer (CPD300; Leica, Wetzlar, Germany). We utilized the carbon conductive paint for mounting. After that, the osmium coating was completed utilizing an Osmium Coater (NL-OPC-AJ, Filgen, Nagoya, Japan). At last, we examined each sample using a microscope (SEM: S-4800; Hitachi High-Technologies Corporation, Tokyo, Japan).

### 2.7. Bacterial Morphology

Bacterial morphological analysis by transmission electron microscopy (TEM) was performed as described below. First, *S. mutans* strains treated with HNS were cultured in TSB at 37 °C for 24 h under 5% CO_2_. We prefixed the samples with 2.5% glutaraldehyde in 0.1 M phosphate buffer (pH 7.4) at 4 °C. After that, we post-fixed the samples with 1% osmium tetroxide in 0.1 M phosphate buffer (pH 7.4) for 45 min. Subsequently, we dehydrated the samples in a graded series of ethanol and embedded them in epoxy resin. We cut ultrathin sections utilizing an ULTRACUT-S (Leica) with a diamond knife and stained them with 2% uranyl acetate in distilled water for 15 min. Then, we stained them with a lead staining solution for 5 min. At last, we evaluated the sections using a JEM-1400 plus electron microscope (JEOL, Tokyo, Japan) at 100 kV. The samples were placed on a 300-mesh carbon formvar copper grid (Nisshin EM), and observed by TEM. We captured the digital images with a Mega View Slow-scan camera (JEOL).

### 2.8. Cell Surface Hydrophobicity

We evaluated the hydrophobicity of the cell surfaces by utilizing the hexadecane method with some modifications [15]. *S. mutans* treated with HNS and those not treated were grown until the exponential phase and adjusted in PBS to 1.0 (A600). After we added the 200 μL of n-hexadecane (Fujifilm Wako) to 2 mL of bacterial suspensions in glass tubes, we measured the A600 value of the lower aqueous phase. Then, the tubes were vortexed for 2 min vigorously, followed by 10 min of incubation at 25 °C for phase separation. After that, we measured the A600 value of the lower aqueous phase. We calculated the hydrophobicity utilizing the following equation: percent of hydrophobicity = (1 − (A600 after vortex/A600 before vortex)) × 100.

### 2.9. Quantitative Real-time RT-PCR

We used RNA extraction by utilizing ISOGEN (Nippon Gene Co. Ltd., Tokyo, Japan) according to the manufacturer’s instructions. Briefly, after culture in TSB with or without HNS at 37 °C for 12 h under 5% CO_2_, the *S. mutans* colonies were disrupted using 300 μL of ISOGEN (Nippon Gene) with glass beads (Sigma-Aldrich, St. Louis, MO, USA). RNA-containing supernatant was treated with 700 μL of ISOGEN for 30 min at 55 °C. After centrifugation, the total RNA was recovered by using chloroform (Fujifilm Wako), isopropanol (Fujifilm Wako), and 70% EtOH (Fujifilm Wako) and dried under appropriate sterile conditions. We quantitated the RNA samples with the Nano Drop ND-1000 (Thermo Fisher). We measured 260/280 nm on this machine to evaluate the RNA. We confirmed that the evaluation value of RNA was all included in 1.8 to 2.0. We synthesized the cDNA with ReverTra Ace qPCR RT Master Mix (Toyobo, Osaka, Japan) according to instructions. We used a total RNA amount of 0.5 μg in this reaction. We used the qPCR for the quantification of cDNA, executed with the Thunderbird qPCR Mix (Toyobo) and Applied Biosystems 7900HT Fast Real Time PCR System (Thermo Fisher) according to the manufacturer’s instructions. The relative quantifications of β-glucosyltransferase (*gtfB*), glucosyltransferase-SI (*gtfC*), and fructosyltransferase (*ftf*) mRNA expression were performed using 16S rRNA as a reference gene. Gene-specific primers were described elsewhere [16]. We also set the PCR program condition as described before [16]. In this assay, we included the appropriate negative and positive controls [16].

### 2.10. Statistical Analysis

We expressed the study data as the means ± standard deviation (SD). We also conducted the statistical analysis using the Student’s *t*-test for 2 groups, and Tukey’s multiple comparison *t*-tests for the differences among multiple groups. The *p*-values < 0.05 indicated statistical significance.

## 3. Results

### 3.1. S. mutans Growth

**Figure 1:**
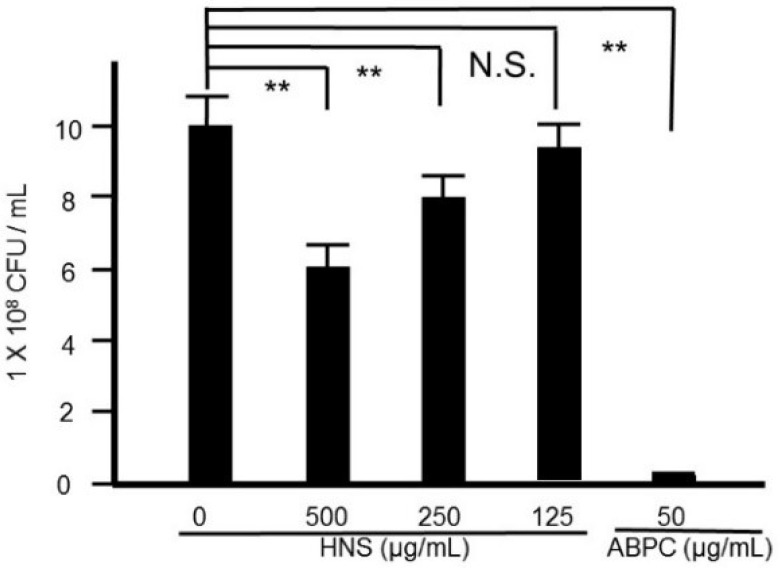
Suppressive effects of the hainosan extract (HNS) on the bacterial growth. *S. mutans* cells were treated with HNS for 24 h. Data points represent the number of viable cells in a culture medium or culture medium containing HNS and are presented as the means ± S.D. (*n* = 6). **: *p* < 0.01 by Tukey’s multiple comparison tests. NS: not significant.

At first, the effect of HNS on the growth of *S. mutans* was investigated. Although we observed significant bacterial reduction by the treatment of HNS in a concentration-dependent manner, HNS did not show a marked bacteriostatic effect as would an antibiotic (Figure 1).

### 3.2. Safranin Red Staining

**Figure 2:**
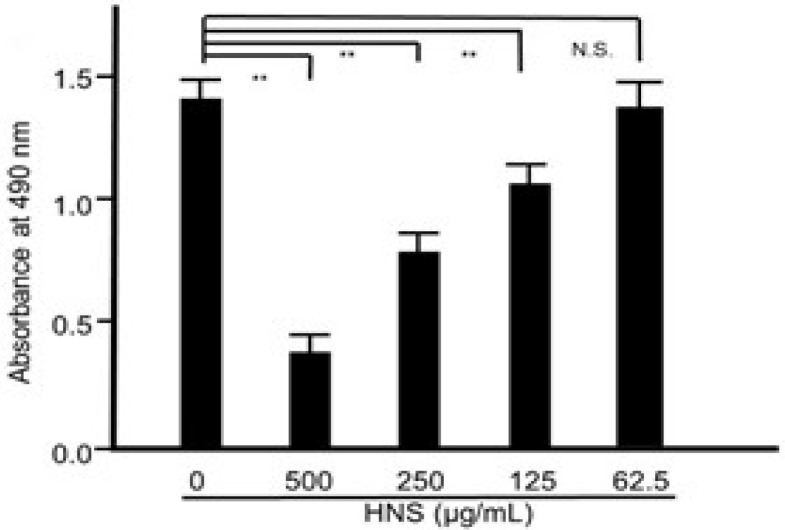
Suppressive effects of the HNS on the biofilm formation by *S. mutans*. *S. mutans* cells were treated with HNS for 48 h. The adherent bacteria were stained with safranin red and the biofilm formation was quantified by absorbance at 490 nm. Data represent the mean ± S.D. (*n* = 6). **: *p* < 0.01 by Tukey’s multiple comparison tests. NS: not significant.

Then, to evaluate whether HNS could suppress biofilm formation, *S. mutans* was cultured in TSB with HNS. As expected, the significant suppressive effect of HNS on *S. mutans* biofilm formation was confirmed (*p* < 0.01) (Figure 2). With an increase in concentration (from 125 μg/mL) of HNS, biofilm formation by *S. mutans* was remarkably inhibited (*p* < 0.01). Thus, we confirmed that the anti-biofilm activity of HNS occurred in a concentration-dependent manner.

### 3.3. Confocal Laser Scanning Microscopy

**Figure 3:**
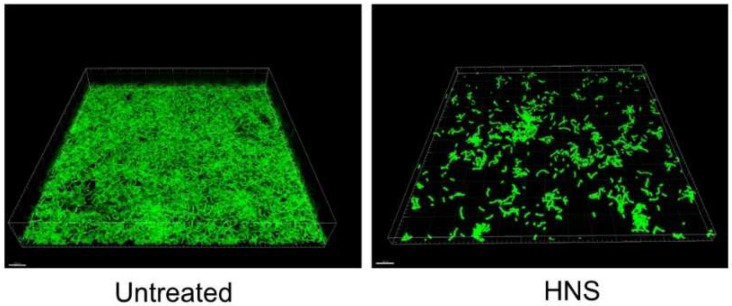
Inhibitory effects of the HNS on the biofilm formation by *S.mutans*, measured by confocal laser scanning microscopy. *S. mutans* cells were treated with HNS (500 µg/mL) for 48 h. The biofilms formed were stained with fluorescein isothiocyanate isomer (FITC), and the three-dimensional findings were obtained from the confocal optical sections.

To verify the data acquired by the safranin red staining, the biofilms formed by *S. mutans* treated with HNS and those not treated were stained with FITC dye. We analyzed by confocal laser scanning microscopy (Figure 3). The acquired Z-stack images were changed into three-dimensional images. The HNS-untreated strains showed a multi-layered surface-attached cluster reflecting a mature biofilm. However, the HNS-treated strains showed a lack of biofilm, including the extracellular matrix. After all, both the results between from the safranin red staining and from the microscopic three-dimensional findings were identical.

### 3.4. Scanning Electron Microscopy (SEM)

**Figure 4:**
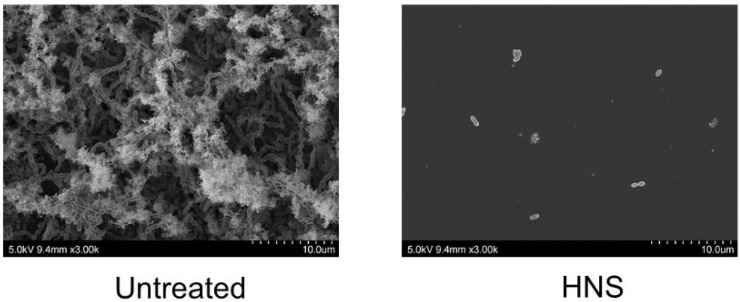
Inhibitory effects of the HNS on the biofilm formation by *S. mutans*, measured by scanning electron microscopy (SEM). *S. mutans* cells were treated with HNS (500 µg/mL) for 48 h. The samples were examined by SEM.

Furthermore, we tried to evaluate another assay using SEM (Figure 4). SEM scanning confirmed the effect of HNS on biofilm formation in glass cover slips. Although *S. mutans* formed a large amount of biofilm in the samples not treated with HNS, the biofilms in those cultured with HNS extract (500 μg/mL) were significantly reduced.

### 3.5. Transmission Electron Microscopy (TEM)

**Figure 5:**
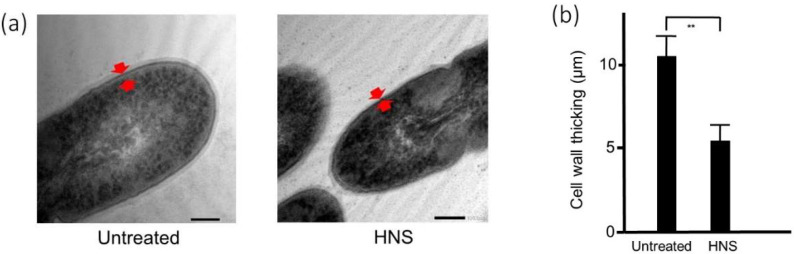
Inhibitory effects of the HNS on the *S. mutans*-formed biofilm, measured by transmission electron microscopy (TEM). *S. mutans* cells were treated with HNS (500 µg/mL) for 24 h. The samples were examined by transmission electron microscopy. (**a**) Representative photo. Red arrow shows the cell wall. (**b**) The thickness was measured at six arbitrary points in the bacteria treated with HNS and in untreated bacteria. Data demonstrate the mean ± SD (*n* = 6). **: *p* < 0.01 by Student’s *t*-test).

To clarify the reason for the reduction of biofilm, we performed the analysis using TEM (Figure 5a). We tried to evaluate the antibacterial effect of HNS on *S. mutans* by morphological analysis. TEM demonstrated that there was no remarkable difference in the bacterial size between HNS-untreated and -treated groups. However, Figure 5 reveals that the cell walls of *S. mutans* treated with HNS were significantly thinner than those of the untreated *S. mutans* (*p* < 0.01)

### 3.6. Hydrophobicity of the Bacterial Cell Surface

**Figure 6:**
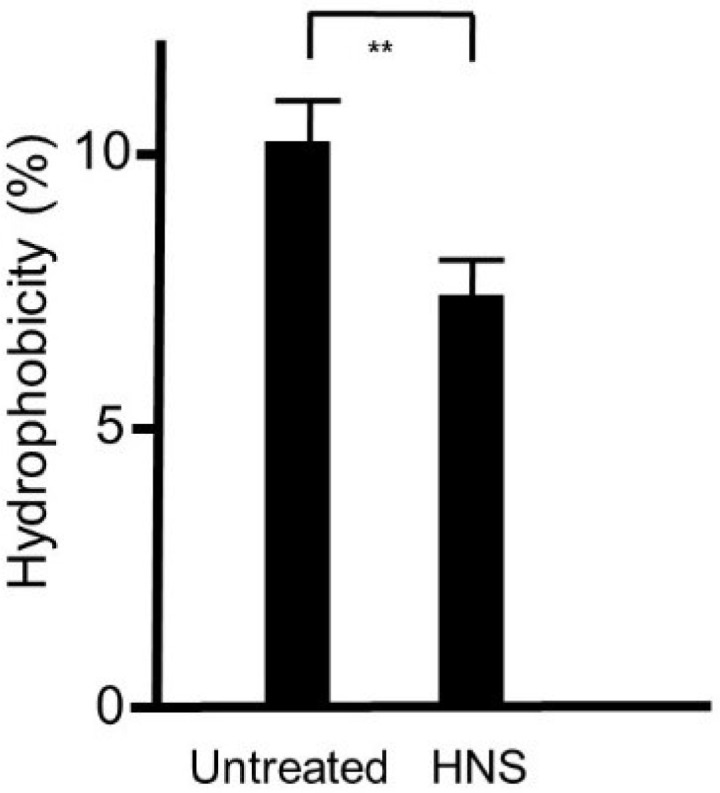
Inhibitory effects of the HNS on the biofilm hydrophobicity of *S. mutans*. *S. mutans* cells were treated with HNS (500 µg/mL) at the exponential phase, and the cell surface hydrophobicity (A600 of 1.0) was determined. The value of the control strain was set at 100%. Data show the mean ± SD (*n* = 6). **: *p* < 0.01 by Student’s *t*-test.

As the hydrophobicity of the bacterial cell surface influences the biofilm formation, we defined the hydrophobicity of the strains utilizing an n-hexadecane method. We showed that the surface hydrophobicity was significantly decreased in *S. mutans* treated with HNS in comparison with that in the *S. mutans* of the untreated group (*p* < 0.01) (Figure 6).

### 3.7. Effects of HNS on Related mRNA Expression of Biofilm Formation

**Figure 7:**
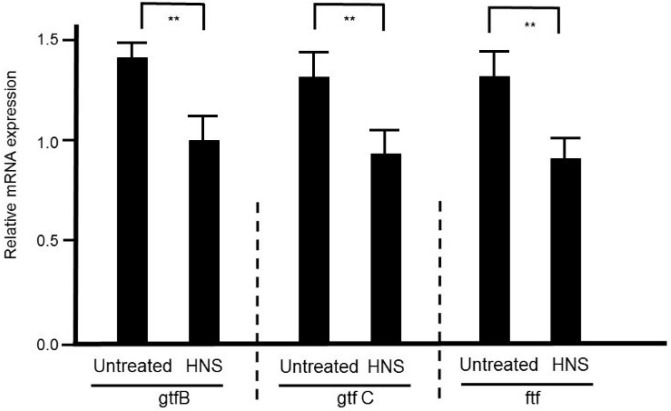
Inhibitory effects of the HNS on the mRNA expression of *β*-glucosyltransferase (gtfB), glucosyltransferase-SI (gtfC), and fructosyltransferase (ftf) in *S. mutans*. *S. mutans* cells were treated with HNS (500 µg/mL) for 12 h or left untreated, and the total RNA was extracted. The relative mRNA values of *gtfB*, *gtfC*, and *ftf* were measured using a RT-PCR system normalized using 16S rRNA as the housekeeping gene. Data represent mean ± S.D. (*n* = 6). **: *p* < 0.01 by Student’s *t*-test.

The mRNA expression of *gtfB*, *gtfC*, and *ftf* in *S. mutans* UA159 was measured using RT-PCR system. Figure 7 shows that the HNS significantly reduced the mRNA levels of these three proteins in comparison with those in the untreated group (*p* < 0.01, respectively). It is indicative of the high efficiency of HNS in the diminution of these adhesive accelerating mRNA expressions in *S. mutans*.

### 3.8. Effect of the Extracts of each Constituent Crude Drug of HNS on S. mutans Biofilm

**Figure 8:**
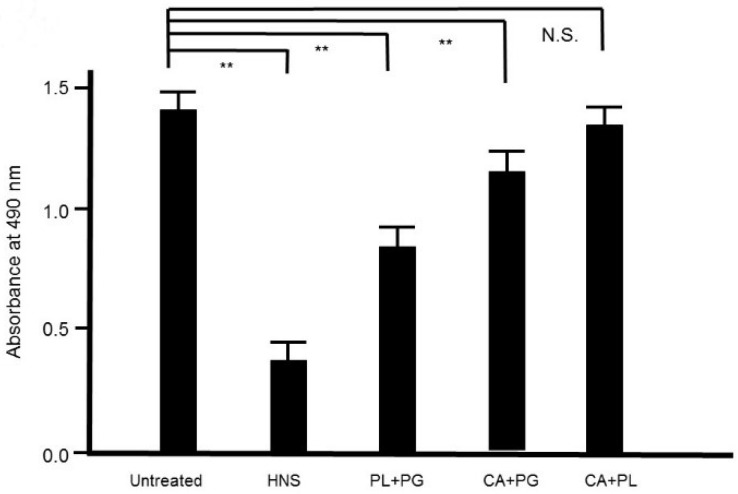
Inhibitory effects of the HNS constituents on the biofilm formation by *S. mutans*. *S. mutans* were treated with the constituents of HNS for 48 h, and stained with safranin red. The concentrations of the samples used were as follows: HNS, 500 µg/mL; extracts of Paeoniae Radix (PL) and Platycodon Radix (PG) mixture, 194 and 127 µg/mL, respectively; extracts of Aurantii Fructus Immaturus (CA) and PG mixture, 179 and 127 µg/mL, respectively; and extracts of CA and PL mixture, 179 and 194 µg/mL, respectively. Data show the mean ± S.D. (*n* = 6). **: *p* < 0.01 by Tukey’s multiple comparison tests. NS: not significant.

**Figure 9:**
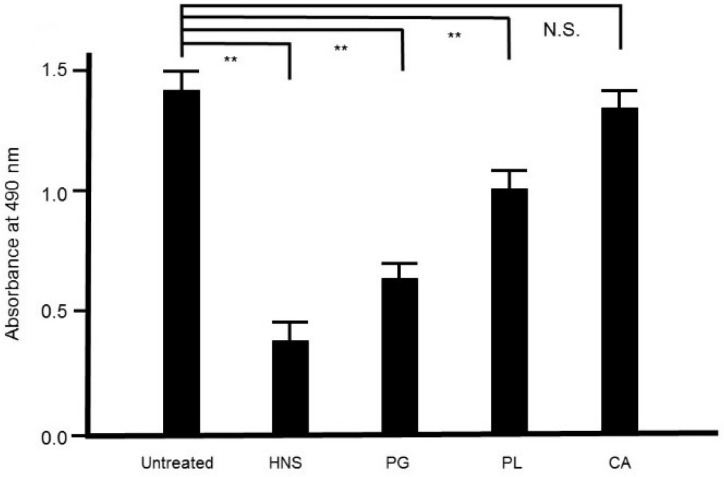
Inhibitory effects of the extracts of the crude drugs composed of the HNS on the biofilm formation by *S. mutans*. Bacteria were treated with the samples for 48 h, then stained with safranin red. The concentrations of the samples used were as follows: HNS, 500 µg/mL; and extracts of Platycodon Radix (PG), Paeoniae Radix (PL), and Aurantii Fructus Immaturus (CA), 127, 194, and 179 µg/mL, respectively. Data demonstrate the mean ± S.D. (*n* = 6). **: *p* < 0.01 by Tukey’s multiple comparison tests. NS: not significant.

To assess the contribution of each crude drug component of HNS to the inhibitory effect on biofilm formation by *S. mutans*, we evaluated the effect of the samples of HNS by removing one component each, using safranin red staining. We found that with removal of PG, HNS did not exhibit any significant suppressive effect on biofilm formation by *S. mutans*. However, significant suppressive effects on biofilm formation by *S. mutans* were exhibited by HNS on removal of the other two crude drugs (*p* < 0.01) (Figure 8). Thus, we speculated that PG played a crucial role in the disruption of bacterial biofilm. To confirm which of hainosan’s constituent crude drugs contributed to the inhibition of biofilm formation by *S. mutans*, we assessed the inhibitory effect of each single crude drug extract on biofilm formation by safranin red staining. As expected, the extract of PG exhibited the highest suppressive effect on *S. mutans* biofilm formation (*p* < 0.01). The extract of PL also showed significant anti-biofilm effects even if weaker (Figure 9).

### 3.9. Effect of Platycodin D on S. mutans Biofilm

**Figure 10:**
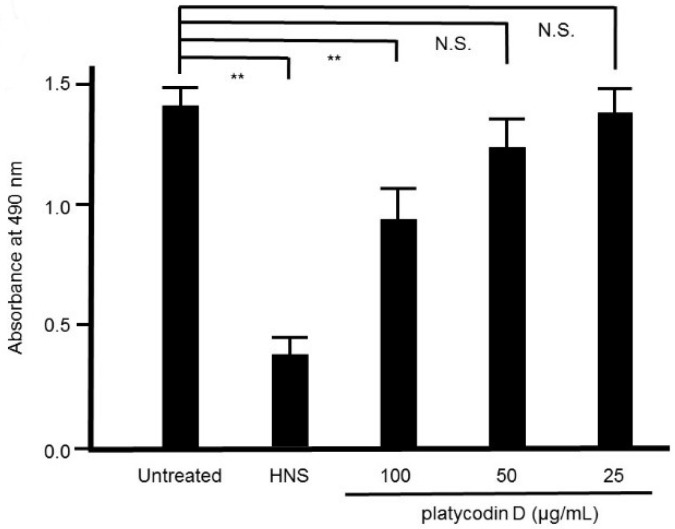
Inhibitory effects of platycodin D on the biofilm formation by *S. mutans*. *S. mutans* were treated with platycodin D for 48 h, and stained with safranin red. Data show the mean ± S.D. (*n* = 3). **: *p* < 0.01 by Tukey’s multiple comparison tests. NS: not significant.

Platycodin D is known as one of the constituents in PG [12]. We assessed the effect of platycodin D on *S. mutans* biofilm formation by safranin red staining. As expected, the remarkable suppressive effect of platycodin D on *S. mutans* biofilm formation was verified in a concentration-dependent manner, and 10 μg/mL of platycodin D remarkably suppressed the biofilm formation by *S. mutans* (*p* < 0.01) (Figure 10).

## 4. Discussion

Here, we clarified the mechanism of the anti-biofilm effect of HNS and its crude drugs on *S. mutans*. We validated that HNS had a biofilm-suppressive effect against *S. mutans* by triple kinds of assays; absorbance, confocal laser microscopy, and scanning electron microscopy. We revealed that this effect of HNS was related to both the disruption of the surface of bacteria and the reduction of hydrophobicity of the surface of bacteria. We also found that the extract of PG, which is a component of HNS, had a biofilm-suppressive effect on *S. mutans*, and that one of its components, platycodin D, also exhibited this effect.

We found that HNS suppressed the expression of *gtfB*, *gtfC*, and *ftf* mRNAs. A fundamental factor of *S. mutans* is its glucan-mediated and sucrose-dependent colonization of tooth surfaces [17]. Extracellular glucosyltransferases synthesize glucans as glucose polymers [18]. As both gtfB and gtfC synthesize principally water-insoluble glucans, gtfD synthesizes water-soluble glucans [19,20]. The gtfs are implicated in cell–cell communications, signal transduction, immune reaction, and microbial attachment [21]. *S. mutans* synthesize extracellular glucans from the glucose moiety of sucrose by gtfB, gtfC, and gtfD and the fructans from the fructose moiety of sucrose by ftf [22]. These *S. mutans*-secreted glucosyltransferases make specific binding sites useful for the bacterial colonization of the tooth surface as the precursor of dental caries [23]. As the product of the ftf gene possesses the function as extracellular storage polysaccharides, it presents a characteristic microenvironment for bacterial life [24]. Thus, the suppression of these polysaccharide synthesis by influencing these encoding genes expression is an effective approach to abrogate the plaque formation and dental caries [24]. As previous studies have shown that the *gtfB*, *gtfC*, and *ftf* genes are critical for the sucrose-dependent adhesion of *S. mutans* to hard surfaces, *gtfD* is not essential [25]. Hence, the *gtfB*, *gtfC*, and *ftf* genes have become attractive targets for protection against tooth decay and we focused on these three biofilm-associated genes in this investigation. As *S. mutans* produce extracellular polysaccharides from dietary sucrose, this bacterial virulent factor remarkably augments the cariogenicity [26]. The less extracellular polysaccharides are generated, the lower the cariogenicity of *S. mutans* [27]. Thus, most researchers are interested in the production and gene regulation of virulence factors, such as gtfs, which are associated with *S. mutans* biofilm, to control tooth decay [23,24]. Although the inhibition of essential virulence factors (gtf and ftf) may be the principal purpose for the prevention of tooth decay and other plaque-related disorders, among the many crude drugs used in traditional Chinese medicine and Kampo medicine, we cannot find any crude drugs that affect glucosyltransferase of *S. mutans* including the suppression of mRNA expression except for those constituting hainosan. We reported the anti-biofilm effect of HNS on *Porphyromonas gingivalis* because of the injury of bacterial cell surface [12]. Although a similar effect on bacteria was seen, we also found a new effect of HNS on suppressing the expression of biofilm-associated genes such as glucosyltransferase in this study. Furthermore, by the removal of PG from HNS and by studying its activity separately, we found that PG contributed to the suppressive effect of bacterial biofilm. Among the chemical ingredients in PG, we identified platycodin D as the active ingredient containing PG. Therefore, the elucidation of the mode of action of HNS, which is commercially available, against not only *P. gingivalis* but also *S. mutans,* will advance our therapeutic selection of anti-biofilm effects.

Although our results only showed a direct effect on bacteria in vitro, which is not a direct reflection of the effect in humans, it is necessary to evaluate how much time and in what concentration the drug can contact bacteria by the oral administration of the drug in the oral cavity. This needs to be confirmed in the future animal studies and human clinical trials.

## 5. Conclusions

Our results showed that the extracts of HNS, especially *Platycodon grandiflorum*, had a suppressive effect on *S. mutans*-induced biofilm. This effect was related to the natural compound, platycodin D, contained in *Platycodon grandiflorum*. Furthermore, the addition of HNS suppressed the biofilm formation of bacteria by reducing the expression of glucosyltransferases and fructosyltransferase of *S. mutans*. We suggested HNS as a promising agent for the elimination of dental plaques.
